# Aldose Reductase Inhibitors of Plant Origin in the Prevention and Treatment of Alcoholic Liver Disease: A Minireview

**DOI:** 10.1155/2019/3808594

**Published:** 2019-06-20

**Authors:** Longxin Qiu, Chang Guo, Baoyu Hua

**Affiliations:** ^1^School of Life Sciences, Longyan University, Longyan 364012, China; ^2^Fujian Province Universities Key Laboratory of Preventive Veterinary Medicine and Biotechnology (Longyan University), Longyan 364012, China; ^3^Fujian Provincial Key Laboratory for the Prevention and Control of Animal Infectious Diseases and Biotechnology, Longyan 364012, China

## Abstract

Alcoholic liver disease (ALD) is caused by heavy alcohol consumption over a long period. Acetaldehyde-mediated toxicity, oxidative stress, and imbalance of lipid metabolism are generally considered involved in the initiation of ALD. There is an increasing requirement for alternative and natural medicine to treat ALD. Recently, aldose reductase (AR) has been reported to be involved in the development of ALD by affecting inflammatory cytokines, oxidative stress, and lipid metabolism. Here, we review the effect of plant-derived AR inhibitors on ALD in rodents. And we conclude that AR inhibitors of plant origin may enhance antioxidant capacity, inhibit lipid peroxidation and inflammatory cytokines expression, and activate AMP-activated protein kinase thereby subsequently suppressing alcohol-induced lipid synthesis in liver to achieve ALD protection. This review reveals that natural AR inhibitor may be potential therapeutic agent for ALD.

## 1. Introduction

Alcoholic liver disease (ALD) is caused by heavy alcohol consumption over a long period. Metabolism of alcohol in the body produces harmful substances, which lead to liver damage and a series of pathological changes in the liver. The incidence of ALD is relatively high among liver diseases. The spectrum of ALD includes alcoholic fatty liver (AFL), alcoholic steatohepatitis (ASH), progressive fibrosis, cirrhosis, and in some cases hepatocellular cancer (HCC) [1]. AFL is an early stage of ALD and it is usually asymptomatic though some patients have hepatomegaly. ASH exhibits symptoms similar to those of chronic hepatitis, such as mild malaise through the entire body, fatigue, exhaustion, upper abdominal discomfort, nausea and vomiting, loss of appetite, and abdominal distension. Moreover, the severe ASH may lead to alcoholic hepatitis (AH), which is an acute clinical presentation of ALD and requires an effective drug treatment strategy. ASH and AH are terms often used interchangeably in scientific literature [2]. Alcohol abstinence is still the best treatment for all stages of ALD. Drug options for AH include corticosteroids as a first choice and pentoxifylline, an inhibitor of phosphodiesterase, as a second line therapy. In the case of advanced disease such as cirrhosis or HCC, liver transplantation may be required [1, 2]. Thus, there is an increasing requirement for alternative and natural medicine to treat ALD. Recently, the aldose reductase (AR)/polyol pathway has been reported to be involved in the development of ALD, and treatment with AR inhibitor improves ALD in rodents [3–5]. This article reviews the biological effect of some potent AR inhibitors of plant origin on ALD, and the mechanisms by which AR inhibitors improve ALD.

## 2. Pathogenesis of ALD

The pathogenesis of ALD has not been fully elucidated. Acetaldehyde-mediated toxicity, oxidative stress, and imbalance of lipid metabolism are generally considered involved in the initiation of ALD [6–9]. The “two-hit” theory has been proposed as a model of the pathogenesis of ALD. Alcohol, acting as the first hit, increases the concentration of reactive oxides by promoting oxidative stress, which induces fat accumulation in the liver; oxidative stress-related lipid peroxidation and inflammatory cytokines act as the second hit on the hepatocytes in fatty liver, causing inflammation, necrosis, and fibrosis [10].

Many studies have suggested that alcohol-induced imbalance in lipid metabolism may be caused by alcohol-induced abnormal expression of genes involved in lipid metabolism, e.g., peroxisome proliferator-activated receptor alpha (PPAR-*α*) or AMP-activated protein kinase (AMPK) expression. PPAR-*α*, a nuclear receptor closely related to lipid metabolism, regulates genes involved in the uptake, binding, transportation, and activation of fatty acids. PPAR-*α* is mainly expressed in hepatocytes and involved in lipid metabolism in the liver. It plays a key role in regulating lipid transport and fatty-acid oxidative degradation in the liver, so it can prevent the development of fatty liver to some extent [11, 12]. Some studies have confirmed the role of PPAR-*α* in the development of ALD [13, 14]. AMPK plays an important role in increasing fatty acid oxidation, enhancing insulin sensitivity, and reducing oxidative stress. It is also closely related to the pathogenesis of ALD [15]. Studies have confirmed that AMPK activity is inhibited in ALD, which weakens the inhibitory effects of acetyl-CoA carboxylase (ACC) and activation of the sterol regulatory element-binding protein (SREBP), resulting in increased lipid synthesis and reduced lipolysis, increased fat accumulation, and the involvement of the “first hit” of ALD pathogenesis. This reduced AMPK expression in ALD may be one of the principal causes of liver damage [16, 17].

The oxidative stress that forms during ethanol metabolism is also an important cause of ALD. Ethanol metabolism increases nicotinamide adenine dinucleotide (NADH) levels and electron flow in the electron transport chain in the mitochondria, activating nicotinamide adenine dinucleotide phosphate (NADPH) oxidase (NOX) and microsomal ethanol oxidase system (MEOS) to generate a large quantity of reactive oxygen species (ROS) [9]. In addition, oxidative stress caused by alcohol metabolism induces lipid peroxidation, resulting in further damage to hepatocytes. The lipid peroxidation caused by oxidative stress induces damage to biological membranes, which reduces the concentration of unsaturated fatty acids in the membrane and causes imbalance in the ratio of unsaturated fatty acids to proteins [6, 9].

During alcohol metabolism, some antioxidant pathways become activated and so interfere with oxidative stress damage in the human body. For example, the endogenous antioxidant superoxide dismutase (SOD) can interfere with alcohol-induced damage in the liver. Antioxidants such as SOD and catalase in the liver and vitamin C in food can eliminate a large proportion of ROS produced after alcohol intake. Long-term heavy consumption of alcohol can cause gastrointestinal dysfunction and reduce the absorption of antioxidants from food sources. Further, the process of alcohol metabolism also consumes large quantities of antioxidants, causing ROS to accumulate in the body [6, 7].

## 3. Aldose Reductase (AR) and ALD

AR is part of the NADPH-dependent aldehyde-keto reductase superfamily. It is multifunctional and acts as a rate-limiting enzyme of the polyol pathway in sugar metabolism. Through the regulation of cytokines, growth factors, oxidative stress, and other mediated intracellular signal transduction pathways, AR participates in a variety of disease pathological processes [48]. AR is widespread in organs and tissues that are related to diabetic complications, such as the kidney, blood vessels, lens of the eye, retina, and heart. It catalytically converts glucose to sorbitol, which cannot easily pass through the cell membrane, resulting in swelling, degeneration, and necrosis of the cells [49]. The roles of AR in the complications of diabetes have been reported extensively in the literature [50, 51].

In addition to being highly expressed in organs and tissues that are relative to diabetic complications, AR expression is also induced in the livers of ALD patients [52] and alcohol-stimulated mice [5]. In a previous study, we showed that AR-inhibitor treatment improved alcohol-induced steatosis in HepG2 cells by activating AMPK and subsequently inhibiting expression of sterol regulatory element binding protein-1c (SREBP-1c) and fatty acid synthase (FAS). It also inhibited alcohol-mediated tumor necrosis factor alpha (TNF-*α*) overexpression to improve alcohol-induced lipid accumulation in hepatocyte [4]. In another previous study, we also showed that AR inhibitor reduced Cytochrome P-450 2E1- (CYP2E1-) mediated oxidative stress and inflammatory cytokine expression, and activated AMPK to improve alcohol-induced hepatic steatosis in mice [5]. Our metabolomics study further demonstrated that AR inhibitor improved alcohol-induced hepatic steatosis in mice by suppressing the biosynthesis of saturated fatty acids [3].

Activation of AR increases the consumption of NADPH and competitively inhibits nitric oxide synthase, which also uses NADPH as a coenzyme. Nitric oxide plays an important role in maintaining the physiological functions of vascular endothelium and nerve tissues. Consumption of large amounts of NADPH causes significant reductions in the amount of nitric oxide synthesized, which ultimately disrupts the reduction-oxidation (redox) system and damages tissue. Increases in NADPH consumption also allow competitive inhibition of the normal synthesis of glutathione (GSH) reductase, which also uses NADPH as a coenzyme, resulting in a significant decrease in the levels of the important antioxidant GSH in cells [53]. Ethanol intake causes damage to the intracellular redox system and a decrease in antioxidant levels, resulting in intracellular oxidative stress and long-term accumulation of fat, thereby leading to ALD [7]. For this reason, it is reasonable to believe that AR plays a vital role in the development of ALD. AR inhibitors may be a suitable resource for the development of drugs for the prevention and treatment of ALD with broad application prospects.

## 4. Role of Plant-Derived AR Inhibitors in the Prevention and Treatment of ALD

**Figure 1:**
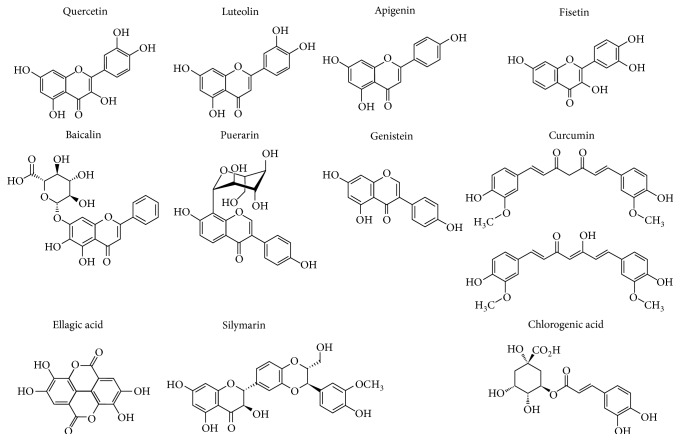
Chemical structure of some potent AR inhibitors of plant origin.

AR inhibitors are generally divided into three categories according to the sources: natural AR inhibitors of plant origin, natural AR inhibitors of microbial origin, and synthetic AR inhibitors such as carboxylic acids, hydantoins, and phenylpyrrolidone compounds. Among the three categories of AR inhibitors, plant AR inhibitors, which mainly include flavonoids, polyphenols, terpenoids, and alkaloids, have seen widespread use because of their wide range of sources and low rate of adverse reactions. Plant-derived compounds with AR inhibitory effect have been extensively reviewed by Veeresham et al. [51] and Kawanishi et al. [54]. Among these compounds, currently only some commercially available ones have been tested for hepatoprotection activity in rodents with ethanol-induced liver injury and are summarized in Table 1 in this review. The AR inhibitory activities of these compounds have been well documented. In a study, more than 90 compounds from several natural medicines and medicinal foodstuffs were examined for their AR inhibitory effect on rat lens AR; among them, quercitrin (IC50=0.15 *μ*M), guaijaverin (IC50=0.18 *μ*M), and desmanthin-1 (IC50=0.082 *μ*M) exhibited potent inhibitory activity on rat lens AR [55]. However, some commercially available flavonoids, including luteolin, quercetin, apigenin, fisetin, and myricitrin, were also found to have strong AR inhibitory activities with IC50 values of 0.45 *μ*M, 2.2 *μ*M, 2.2 *μ*M, 3.7 *μ*M, and 3.8 *μ*M respectively, although their activities were weaker than epalrestat, a commercial synthetic aldose reductase inhibitor that inhibits AR with an IC50 of 0.172 *μ*M. In another study, a phenolics, ellagic acid, isolated from* Chrysanthemum morifolium* exhibited inhibitory activity with an IC50 of 0.2 *μ*M, which is stronger than that of quercetin (1.2 *μ*M) in this study [56]. These studies demonstrate that luteolin, quercetin, apigenin, rhamnetin, fisetin, myricitrin, and ellagic acid are potent AR inhibitors. Moreover, silybin, puerarin, and baicalin were found to exhibit markedly inhibitory effects on AR comparable to sorbinil, another commercial synthetic aldose reductase inhibitor [57]. In addition, genistein, puerarin and baicalin were reported to inhibit rat lens AR with IC50 values of 4.5 *μ*M, 44.7 *μ*M, and 25.1 *μ*M, respectively [58]. And curcumin inhibits AR with an IC50 of 10 *μ*M in a noncompetitive manner in one study [59], or with an IC50 of 6.8 *μ*M in the other study [60]. Chlorogenic acid, another phenolic compound, was also reported to inhibit rat AR with an IC50 of 0.95 *μ*M [61]. These studies demonstrate that luteolin, quercetin, apigenin, fisetin, myricitrin, genistein, silymarin/silybin, ellagic acid, curcumin, and chlorogenic acid are potent AR inhibitors. The chemical structures of these compounds are depicted in Figure 1.

Previously we demonstrated that zopolrestat, a chemically synthesized AR inhibitor, improved ethanol-induced liver injury in mice through suppressing oxidative stress, reducing proinflammatory cytokines expression and modulating AMPK/SREBP-1c pathway to attenuate fatty acid synthesis [5]. The same mechanisms whereby zopolrestat improves lipid accumulation were also demonstrated in ethanol-intoxicated mouse AML12 liver cells [5] and human hepatoma HepG2 cells [4]. Of note, among the plant-derived AR inhibitors which were found to exert hepatoprotection effect in rodents, all of them were at least in part through potential antioxidant and anti-inflammatory mechanisms. A recent work reported that chlorogenic acid ameliorated ethanol-induced reactive oxygen species and subsequently reduced hepatic steatosis in mice [47]. Moreover, luteolin, quercetin, apigenin, fisetin, and curcumin were demonstrated to alleviate hepatic steatosis through modulating AMPK/SREBP-1c pathway. Besides these in vivo studies, some in vitro studies also demonstrated that AR inhibitors from nature, including myricitrin [62], puerarin [63], and isoliquiritigenin [64], protected ethanol-intoxicated hepatocytes by regulating the AMPK signaling pathway. In addition, phenolic acid and flavonoid rich ethyl acetate fraction of* S. quelpaertensis* extract (SQEA) was reported to exert cytoprotective effect against ethanol-induced toxicity in HepG2 cells and exhibit antisteatosis effect via the activation of AMPK signaling pathway in alcohol challenged livers [65]. Interestingly, p-coumaric acid and rutin, myricetin were the predominant phenolic acid and flavonoid in SQEA, and they are all AR inhibitors. These studies together demonstrate that AR inhibitors from plant protect against alcohol-induced liver injury through the similar mechanisms with those of chemical synthesized AR inhibitor and indicate that AR inhibitors from nature may be a promising source for developing anti-ALD drugs.

## 5. Conclusion

**Figure 2:**
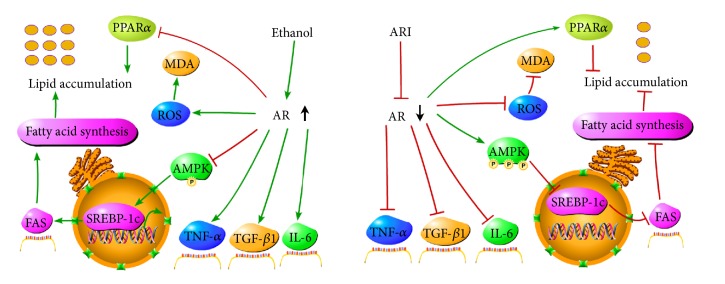
Schematic showing the mechanisms by which aldose reductase inhibitors prevent ALD. AR, aldose reductase; ARI, AR inhibitors; AMPK, AMP-activated protein kinase; SREBP-1c, sterol regulatory element binding protein-1c; FAS, fatty acid synthase.

In summary, as depicted in Figure 2, AR participates in the development of ALD by affecting inflammatory cytokines, oxidative stress, and lipid metabolism in liver while natural AR inhibitors may reduce inflammation, enhance antioxidant activation, inhibit lipid peroxidation, and suppress alcohol-induced lipid synthesis through activating AMPK in liver to achieve ALD protection. This review provides a theoretical basis for the use of natural AR inhibitors in the prevention and treatment of ALD.

## Figures and Tables

**Table 1:** Effects of some natural AR inhibitors on ALD in rodents.

Treatment	Experimental model	Effects	Ref.
Quercetin	Rats treated with 50% ethanol for 10 days	Prevents ethanol-induced liver injury by enhancing antioxidative activity and suppressing the induction of cytokines, chemokines	Chen *et al*. [18]
Quercetin	Rats administrated with alcohol (4.0 g/kg) for 90 days	Protects against chronic ethanol toxicity through its hypolipidemic effect and antioxidative role	Tang *et al*. [19]
Quercetin	Rats administrated with alcohol (4.0 g/kg) for 90 days	Attenuates ethanol-derived microsomal oxidative stress by suppressing the downregulation of HO-1 and the induction of CYP2E1	Tang *et al*. [20]
Quercetin	Rats treated with ethanol (2.0 g/kg) for 30 days	Prevents long-term alcohol consumption-induced oxidative stress and cytokines	Kahraman *et al*. [21]
Quercetin	Mice fed with Lieber-deCarli alcohol-liquid diets for 15 weeks	Alleviates ethanol-elicited mitochondrial damage through enhancing AMPK- and ERK2-mediated mitophagy	Yu *et al*. [22]
Luteolin	Mice exposed to alcohol (1%, 2%, and 4% for 3 d, and 5% for 9 d) and a binge (30% ethanol) on the last day	Ameliorates ethanol-induced hepatic steatosis and injury by activating AMPK and suppressing SREBP-1c/FAS pathway	Liu *et al*. [23]
Apigenin	Mice given 56% erguotou wine by gavage for 30 days	Exerts a protective effect on alcohol-induced liver injury by regulating hepatic CYP2E1-mediated oxidative stress and PPAR*α*, SREBP-1c and FAS gene expression	Wang *et al*. [24]
Fisetin	Mice given 50% ethanol p.o. (10 ml/kg body weight) every 12 hours for a total of 5 doses	Ameliorate alcohol-induced hepatic damage by restoring the antioxidant and MMP/TIMP balance	Koneru *et al*. [25]
Fisetin	Mice fed with Lieber-deCarli alcohol-liquid diets for 4 weeks	Attenuates alcohol-induced hepatic steatosis by increasing hepatic protein levels of p-AMPK, ACOX1, CYP4A, and MTTP	Sun *et al*. [26]
Baicalin	Rats intragastrically administrated with alcohol continuously for 4 or 8 weeks	Exerts beneficial effects on alcohol-induced liver injury through inhibiting oxidative stress, proinflammatory cytokines expression, and the regulation of the sonic hedgehog pathway	Wang *et al*. [27]
Baicalin	Mice treated by chronic plus binge ethanol feeding	Ameliorates ethanol-induced liver injury by modulating oxidative stress and inflammation via CYP2E1 and NRF2	He *et al*. [28]
Puerarin	Rats treated with 40% ethanol (8 g/kg/d) for 5 days	Prevents acute ALD by enhancing antioxidative capacity	Zhao *et al*. [29]
Puerarin	Rats provided with the Liber-deCarli liquid diet for 8 weeks	Alleviates chronic alcoholic liver injury by inhibiting endotoxin gut leakage, Kupffer cell activation, and endotoxin receptors expression	Peng *et al*. [30]
Puerarin	Rats treated with 6 g/kg/d, 7 g/kg/d, 8 g/kg/d (for a period of 1 week respectively), and 9 g/kg/d (for a period of 21 weeks) of 56% alcohol	Protects against alcohol-induced liver lesions through improving metabolic function	Chen *et al*. [31]
Puerarin/ Genistein	Mice gastrically infused with 50% alcohol once per day for 5 weeks	Alleviates hepatic damage induced by chronic alcohol administration through potential antioxidant, anti-inflammatory, or anti-apoptotic mechanisms	Zhao *et al*. [32]
Genistein	Rats underwent intragastric administration of alcohol (5.0–9.5 g/kg) once a day for 24 weeks	Ameliorates ethanol-induced liver injury and even liver fibrosis by decreasing oxidative stress and production of inflammatory and by inhibiting fibrogenic mediators	Huang *et al*. [33]
Curcumin	Rats treated with ethanol (starting dose was 8 g/kg/d and final dose was 16 g/kg/d) plus fish oil for 4 weeks	Prevents experimental ALD by suppressing the activation of NF-*κ*B and the induction of cytokines, chemokines	Nanji *et al*. [34]
Curcumin	Mice treated with ethanol (2.4 g/kg/day ethanol for the initial 4 weeks and 4 g/kg/day for another 2 weeks)	Prevents chronic ALD by decreasing ROS generation and enhancing antioxidative capacity	Rong *et al*. [35]
Curcumin	Mice administered orally with alcohol (5 g/kg body weight) once a day for 6 weeks and fed a high-fat diet	Protects alcohol-induced liver damage by modulating alcohol metabolic pathway, enhancing antioxidant activity and activating AMPK	Lee *et al*. [36]
Curcumin	Rats given ethanol (56% v/v, 10 mL/kg) orally once every day for 9 weeks	Attenuates ALD by modulating lipid deposition in hepatocytes via a Nrf2/FXR activation and modulating the expression of SREBP-1c, fatty acid synthase, and PPAR-*α*	Lu *et al*. [37]
Curcumin	Mice given 2.4 g/kg/day ethanol plus olive oil once a day for 6 weeks	Protects the liver from chronic-ethanol induced injury through attenuating oxidative stress, at least partially, through ERK/p38/Nrf2-mediated anti-oxidant signaling pathways	Xiong *et al*. [38]
Curcumin	Rats fed with Lieber-deCarli low menhaden and high menhaden alcohol-liquid diets for 8 weeks	Protects against chronic alcohol-induced liver injury by enhancing antioxidative capacity	Varatharajalu *et al*. [39]
Curcumin	Mice fed with Lieber-deCarli alcohol-liquid diets for 4 weeks	Improves alcoholic fatty liver by inhibiting biosynthesis of unsaturated fatty acids, fatty acid biosynthesis and pentose and glucuronate interconversions	Guo *et al*. [40]
Curcumin	Rats fed 50% ethanol (7.5 g/kg body weight/day) orally twice a day for 4 weeks	Improves ethanol-induced liver injury by reducing oxidative stress and inhibiting NF-*κ*B activation	Samuhasaneeto *et al*. [41]
Ellagic acid	Rats fed 20% alcohol orally (7.9 g/kg body weight) for 45 days	Exerts beneficial effects against alcohol-induced damage	Devipriya *et al*. [42]
Ellagic acid	Rats fed 20% alcohol orally (7.9 g/kg body weight) for 45 days	Decreases the expression pattern of fibrotic markers during alcohol-induced toxicity	Devipriya *et al*. [43]
Ellagic acid	Mice fed with Lieber-deCarli alcohol-liquid diets for 5 weeks	Improves alcoholic fatty liver by suppressing the expression of the genes related to cell stress and up-regulating the genes involved in bile acid synthesis, unsaturated fatty acid elongation, and tetrahydrofolate synthesis	Yao *et al*. [44]
Silymarin /Silybin	Mice received ethanol (5 g/kg body weight) by gavage every 12 hours for a total of 3 doses	Protects against the acute alcoholic liver injury by decreasing oxidative stress and production of inflammatory cytokines	Song *et al*. [45]
Silymarin /Silybin	Mice fed ethanol (1.6 g/kg body weight) for 12 weeks	Prevents long-term alcohol consumption-induced liver injury by enhancing antioxidant activity and suppressing the induction of cytokines	Das *et al*. [46]
Chlorogenic acid	Mice fed ethanol (3 g/kg body weight) for 7 consecutive days	Prevents ethanol-induced acute liver injury by reducing oxidative stress, steatosis, apoptotic cell death, and fibrosis	Kim *et al*. [47]
